# Adrenal-Derived Hormones Differentially Modulate Intestinal Immunity in Experimental Colitis

**DOI:** 10.1155/2016/4936370

**Published:** 2016-06-14

**Authors:** Patrícia Reis de Souza, Helioswilton Sales-Campos, Paulo José Basso, Viviani Nardini, Angelica Silva, Fernanda Banquieri, Vanessa Beatriz Freitas Alves, Javier Emílio Lazo Chica, Auro Nomizo, Cristina Ribeiro de Barros Cardoso

**Affiliations:** ^1^Departamento de Análises Clínicas, Toxicológicas e Bromatológicas, Faculdade de Ciências Farmacêuticas de Ribeirão Preto, Universidade de São Paulo, 14040-903 Ribeirão Preto, SP, Brazil; ^2^Departamento de Bioquímica e Imunologia, Faculdade de Medicina de Ribeirão Preto, Universidade de São Paulo, 14040-903 Ribeirão Preto, SP, Brazil; ^3^Instituto de Ciências Biológicas e Naturais, Universidade Federal do Triângulo Mineiro, Uberaba, MG, Brazil

## Abstract

The adrenal glands are able to modulate immune responses through neuroimmunoendocrine interactions and cortisol secretion that could suppress exacerbated inflammation such as in inflammatory bowel disease (IBD). Therefore, here we evaluated the role of these glands in experimental colitis induced by 3% dextran sulfate sodium (DSS) in C57BL/6 mice subjected to adrenalectomy, with or without glucocorticoid (GC) replacement. Mice succumbed to colitis without adrenals with a higher clinical score and augmented systemic levels of IL-6 and lower LPS. Furthermore, adrenalectomy negatively modulated systemic regulatory markers. The absence of adrenals resulted in augmented tolerogenic lamina propria dendritic cells but no compensatory local production of corticosterone and decreased mucosal inflammation associated with increased IFN-*γ* and FasL in the intestine. To clarify the importance of GC in this scenario, GC replacement in adrenalectomized mice restored different markers to the same degree of that observed in DSS group. Finally, this is the first time that adrenal-derived hormones, especially GC, were associated with the differential local modulation of the gut infiltrate, also pointing to a relationship between adrenalectomy and the modulation of systemic regulatory markers. These findings may elucidate some neuroimmunoendocrine mechanisms that dictate colitis outcome.

## 1. Introduction

The immune and endocrine systems interact directly to maintain the homeostasis of the organism in face of aggressions such as stress, infectious diseases, or inflammatory processes. In this context, chronic stress may represent a potential risk factor for the development of autoimmune and inflammatory disorders such as inflammatory bowel diseases (IBD) [1]. IBD that comprise Crohn's disease (CD) and ulcerative colitis (UC) are characterized by their chronic course with alternating episodes of disease activity, severity, and clinical remission [2, 3]. UC and CD are believed to be multifactorial disorders [4] triggered by disturbances in environmental factors (microbiota and stress) [3, 5], genetic susceptibility, and immunological imbalance [4]. Hence, gut dysbiosis [6], defects in the population of effector T cells that react against normal microbial antigens in the intestine, and a decrease in the population of regulatory T cells (Tregs) may account for the breakdown of mucosal tolerance in this scenario [7].

The endocrine system may also play an important immune regulatory role in inflammatory diseases by production of mediators such as the adrenal-derived hormones [8]. During homeostasis disturbance, the secretion of proinflammatory cytokines by immune cells stimulates the hypothalamus to synthesize corticotropin-releasing hormone (CRH), which in turn acts on the anterior pituitary promoting the production and release of adrenocorticotropin hormone (ACTH). The subsequent activation of the adrenal glands by ACTH limits inflammatory responses by systemic release of endogenous glucocorticoids (GCs) [8–10]. GCs are steroid hormones with potent anti-inflammatory activity, produced mainly by the adrenal glands after activation of the hypothalamic-pituitary-adrenal (HPA) axis, in response to various stimuli such as emotional, physical, and/or immune stress [11]. In this context, the removal of the adrenal glands or the systemic pharmacological inhibition of GCs synthesis can result in shock and death after induction of a strong immune response [12]. Moreover, hyporesponsiveness of the HPA axis to stress has been related to the development and perpetuation of inflammation [13, 14]. Furthermore, besides the variable efficacy of exogenous GCs in the treatment of IBD, the effect of adrenal-derived GCs in the modulation of immune effector responses during gut inflammation is still unknown, as well as the relationship between these steroid hormones with regulatory or tolerogenic profiles in the disease, especially in the intestine.

Thus, since the mechanisms by which the adrenal glands modulate inflammatory responses have not been fully elucidated yet, in this study we evaluated the role of these glands and endogenous GC in the regulation of the exacerbated inflammation during experimental colitis.

## 2. Material and Methods

### 2.1. Animal Studies

All studies were performed in accordance with the Guide for the Care and Use of Laboratory Animals (2011 (8th ed.), Washington, DC: National Research Council, National Academies Press) [15] and approved by the Institutional Animal Care and Use Committee of the University of São Paulo (Brazil), under protocol 11.1.522.53.0. Male C57BL/6 mice, aged 6–8 weeks, weight 20–25 g, were maintained under controlled temperature (25°C), in specific pathogen-free and standard controlled environmental conditions with a 12 h light/dark cycle, with food and water* ad libitum* in the animal housing facility of the School of Pharmaceutical Sciences of Ribeirão Preto, University of São Paulo. The experiments were performed with 5 mice/group, and groups were arranged as follows:* Control*: healthy mice without colitis; DSS, animals exposed to water containing dextran sulfate sodium (DSS) to colitis induction; ADX, adrenalectomized mice, without induction of colitis; ADX + DSS, adrenalectomized mice, exposed to DSS; ADX + DSS + GC, adrenalectomized mice, exposed to DSS and treated with 1 mg of dexamethasone/Kg/day, from the 3rd to 5th day of DSS exposure and* Sham*, Sham operated mice. As the main results of the groups* Control*, ADX and* Sham* did not differ significantly from each other in the main assays; we used data of* Control* mice for comparisons in all figures.

### 2.2. Bilateral Adrenalectomy

Bilateral adrenalectomy was performed via a dorsolateral surgery after anesthesia with ketamine (100 mg/Kg) and xylazine (10 mg/Kg) (Agener União Saúde Animal, SP, Brazil). The incisions were closed with absorbable suture thread (Chrome Catgut Absorbable, 5-0, Bioline Fios Cirúrgicos Ltda., GO, Brazil). After surgery, in order to compensate loss of mineralocorticoid hormones because of adrenal glands removal, mice received daily saline solution 0.9% by gavage until the time of euthanasia. On the first 48 hours after surgery animals were also given the nonsteroidal anti-inflammatory drug meglumine flunixin (Banamine®, Schering-Plough Animal Health, NJ, USA) at 1 mg/kg 12/12 hours, subcutaneously. Colitis was induced by DSS 15 days after surgery recovery.

### 2.3. DSS-Induced Colitis, Clinical Assessment, and Macroscopic Analysis

Colitis was induced by 3% dextran sodium sulfate (DSS-MP Biomedicals, Illkirch, France. Molecular weight: 36,000–50,000 kDa) added to the drinking water during 6 days for sample collection. Mice were evaluated daily for body weight change and clinical signs of disease to summarize a clinical disease score, as previously described [16]. The macroscopic analysis of the colon at the day of euthanasia was performed to quantify the “postmortem score,” as described by Sales-Campos et al., 2015 [16].

### 2.4. Glucocorticoid Replacement and Euthanasia

Dexamethasone (Azium® Solução, Coopers Saúde Animal, RJ, Brazil) was dissolved in saline and administered by intraperitoneal (i.p.) route at a dose of 1 mg/kg/day, according to previous dose-response experiments performed by our group. The treatment with GC was held from the 3rd to the 5th day of DSS exposure and euthanasia was performed on the 6th day of colitis. To note, GC treatment began when at least one mouse/cage displayed clinical signs of disease, which means day 3 after DSS exposure.

### 2.5. Histopathological Analysis

The large intestine samples were fixed in PBS/10% formaldehyde followed by standard histology procedures and paraffin embedding. Microtomy was performed to obtain 5 micrometers' (*µ*M) thick sections that were later stained by hematoxylin and eosin (H&E) for microscopic analysis of tissue inflammation. The criteria for the diagnosis of inflammatory bowel disease were adapted from guidelines of the British Society of Gastroenterology [17].

### 2.6. Quantification of Cytokines by Enzyme Linked Immunosorbent Assay (ELISA)

The presence of the cytokines IL-6 and IFN-*γ* was assessed by ELISA according to the manufacturer's instructions (BD Biosciences, San Jose, CA, USA) on sera or gut homogenates containing protease inhibitors (Complete®, Roche, Pharmaceuticals, Mannheim, Germany). FasL dosage in homogenates of colon samples was performed according to the manufacturer's instructions (R&D Systems, Inc., Quantikine®, ELISA Mouse Fas Ligand/TNFSF6 Immunoassay, USA and Canada).

### 2.7. Detection of Corticosterone Synthesis in Intestine Cultures and Plasma

In specific experiments, colon fragments were removed and cultured for quantification of tissue corticosterone as described by Noti et al. [18, 19]. The culture supernatant was assayed by competition ELISA according to the manufacturer's instructions (Corticosterone EIA kit, Cayman Chemical Company, Ann Arbor, MI, USA). Results were expressed as nanograms of corticosterone per gram of tissue and were shown as the difference of samples cultured without metyrapone and samples cultured with metyrapone to correct for putative contamination with serum GC. Similarly, the plasma concentration of corticosterone was determined by the competitive ELISA in blood samples collected in EDTA tubes. The results were expressed as picograms of corticosterone per milliliter (pg/mL).

### 2.8. Isolation of Leukocytes from Spleen, Mesenteric Lymph Nodes, Intraepithelial Compartment, and Lamina Propria

Spleen and MLNs were removed and macerated through a 70 *μ*m cell strainer (BD Biosciences, Heidelberg, Germany). Red blood cells were lysed using ACK (ammonium-chloride-potassium) buffer. The colon leukocytes were isolated according to previous studies [20, 21]. All cell suspensions were tested for viability by Trypan blue dye at 0.2% in a Neubauer chamber before being used in the culture and/or immunophenotyping experiments.

### 2.9. Flow Cytometry

For phenotypic characterization of the population of leukocytes from spleen, MLN, LP, or IEL, monoclonal antibodies (BD Pharmingen*™*, San Diego, USA) conjugated to fluorochromes were used, according to the manufacturer's instructions. Cells were acquired on DIVA Flow Cytometer (BD Biosciences, San Jose, CA) and data obtained was analyzed using FlowJo version 7.6.3 software (TreeStar, San Carlos, CA).

### 2.10. Serum Endotoxin (LPS) Levels

Serum LPS was quantified with a commercially available* limulus amebocyte lysate* assay (QCL-1000, Lonza, Walkersville, MD), according to the manufacturer's protocol. The results were expressed as EU/mL (endotoxin units/milliliter).

### 2.11. Statistics Analysis

In all variables the normal distribution and homogeneous variance were tested. When the distribution was normal and there was homogeneous variance we used the parametric tests ANOVA with Tukey's multiple comparison. In case of non-Gaussian distribution we used the nonparametric tests Kruskal-Wallis, followed by Dunn's multiple comparison test. Results were expressed as mean ± standard error of the mean. Differences were considered statistically significant when *p* < 0.05 (5%). All analyses were performed using the GraphPad Prism 5.0 software (San Diego, CA).

## 3. Results

### 3.1. Adrenal Glands Are Important to Control Clinical Outcome in Experimental Colitis

**Figure 1:**
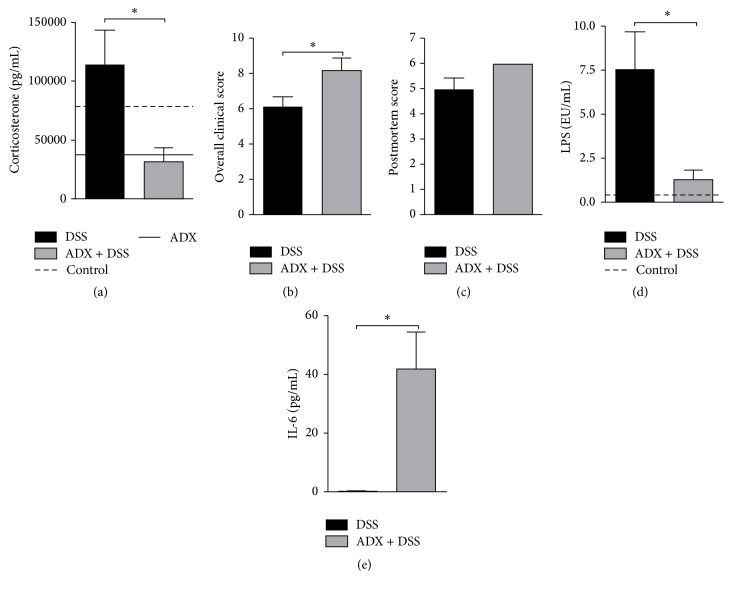
Adrenal glands are important to control clinical outcome in experimental colitis. C57BL/6 mice were adrenalectomized and exposed to dextran sulfate sodium (DSS) 3%. On day 6 mice were euthanized to obtain samples of serum: (a) plasma corticosterone levels; (b) overall clinical score of the disease; (c) postmortem score; (d) serum IL-6 levels; (e) serum lipopolysaccharide (LPS) levels. DSS: C57BL/6 mice exposed to DSS; ADX + DSS: C57BL/6 adrenalectomized mice exposed to DSS. The dashed lines correspond to the control group of C57BL/6 mice not exposed to DSS. Continuous line in (a) corresponds to adrenalectomized mice without colitis. EU/mL = endotoxin units/mL. These results are representative of 3 independent experiments with 5 mice/group. ^*∗*^ *p* < 0.05.

First, to verify if the adrenals or their products could influence colitis clinical outcome, we evaluated mice after DSS exposure in the presence or absence of the glands. The results showed that the clinical signs that peaked on day 6 in nonadrenalectomized mice (data not shown) were accompanied by increased plasma corticosterone levels (Figure 1(a)), which probably resulted from adrenal glands activation during the development of intestinal inflammation. However, as expected, there was a reduction in circulating corticosterone after adrenalectomy (Figure 1(a)), simultaneously to an increased overall disease score (Figure 1(b)) with no significant augmented postmortem score (Figure 1(c)). In addition, since intestinal disruption with DSS leads to bacteria translocation and systemic inflammation, we detected elevated IL-6 cytokine in serum (Figure 1(d)), together with a significant reduced endotoxemia in adrenalectomized mice exposed to DSS on day 6 (Figure 1(e)), suggesting an opposite role between systemic inflammatory responses and reduced LPS levels during colitis induction, in the absence of adrenal glands.

### 3.2. Reduced Accumulation of Regulatory T Cells in the Absence of Adrenal Glands

**Figure 2:**
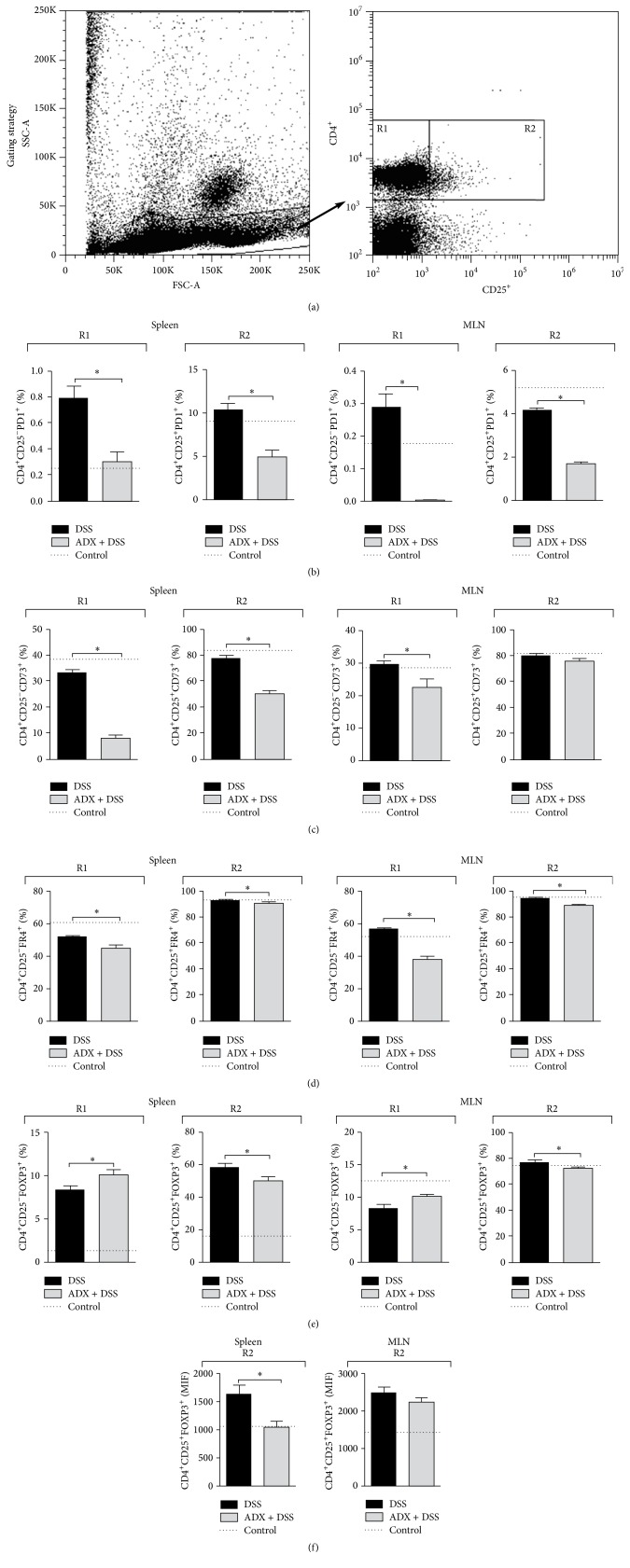
Reduced accumulation of regulatory T cells (Treg) in the absence of adrenal glands. C57BL/6 mice were adrenalectomized and exposed to dextran sulfate sodium (DSS) 3%. The spleen and MLN (mesenteric lymph nodes) were collected on the 6th day of colitis and processed for staining with specific antibodies for regulatory markers and flow cytometry. Analyses were performed according to the cell size (FSC) and granularity (SSC), followed by a selecting gate in the lymphocytes region and evaluation of dot plots for CD4^+^CD25^+^ and CD4^+^CD25^−^ cells (a), in which the frequency of Treg markers was quantified by histograms. (b) Percentage of CD4^+^CD25^−^PD1^+^ and CD4^+^CD25^+^PD1^+^ leukocytes from spleen and MLN. (c) Frequency of CD4^+^CD25^−^CD73^+^ and CD4^+^CD25^+^CD73^+^ cells from spleen and MLN. (d) Percentage of CD4^+^CD25^−^FR4^+^ and CD4^+^CD25^+^FR4^+^ leukocytes from spleen and MLN. (e) Frequency of CD4^+^CD25^−^FoxP3^+^ and CD4^+^CD25^+^FoxP3^+^ cells from spleen and MLN. (f) Mean fluorescence intensity (MFI) of FoxP3 in the CD4^+^CD25^+^ population of spleen and MLN. DSS: C57BL/6 mice exposed to DSS; ADX + DSS: C57BL/6 adrenalectomized mice exposed to DSS. The dotted lines correspond to the control group without colitis. Results are representative of two independent experiments, with 5 mice/group. ^*∗*^ *p* < 0.05.

Once adrenal-derived hormones, including glucocorticoids, are associated with suppression of immune responses, we next evaluated if the systemic inflammation was accompanied by alterations in the pool of regulatory T cells (Tregs) in colitis adrenalectomized mice. Indeed, most of the regulatory markers such as PD-1 (Figure 2(b)), CD73 (Figure 2(c)), and FR4 (Figure 2(d)) were reduced in both CD4^+^CD25^+^ and CD4^+^CD25^−^ populations of these mice, in the spleen and mesenteric lymph nodes (MLN), except for CD4^+^CD25^+^CD73^+^ in the MLN. The CD4^+^CD25^+^FOXP3^+^ cells, which characterize the main natural Treg population, were also reduced by adrenalectomy (Figure 2(e)), along with an increase in the CD4 lymphocytes expressing FOXP3 in the absence of CD25. Most interestingly, there was a clear reduction in the mean fluorescence intensity (MFI) of FOXP3 in the spleen (Figure 2(f)), indicating that the systemic inflammation in the absence of adrenals could be the result of a lack of natural suppressive mechanisms in the host. It is of note that these mice also had reduced frequency of CD3^+^CD49b^+^ lymphocytes (supposedly NKT cells) in the spleen (and not in the MLN), corroborating the findings of compromised systemic regulation following adrenalectomy (data not shown).

### 3.3. Gut Responses Are Differentially Modulated by Adrenal Glands

**Figure 3:**
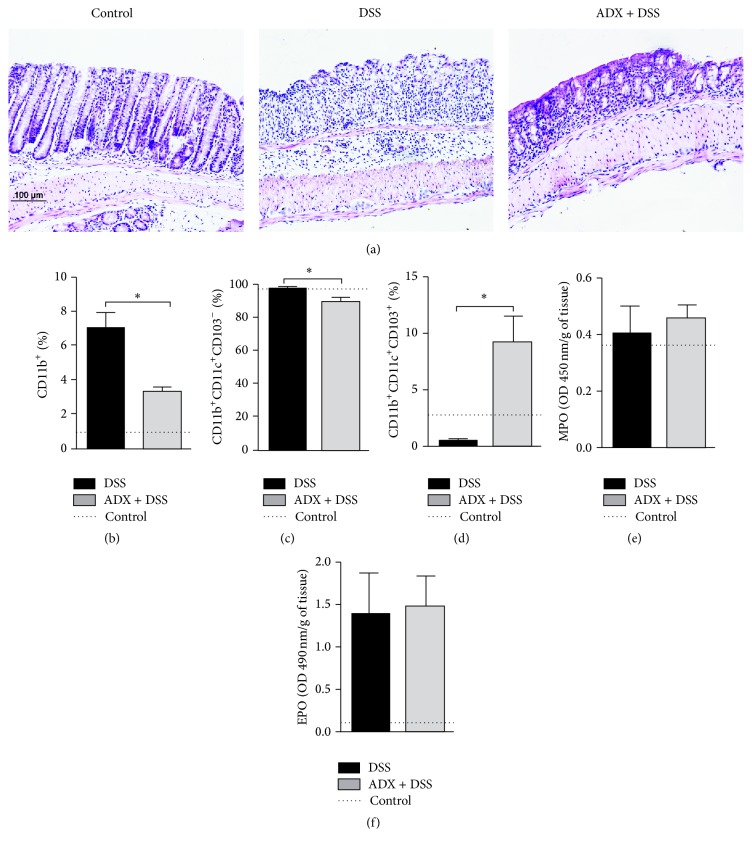
Adrenal-derived mediators modulate local tissue damage. C57BL/6 mice were adrenalectomized and exposed to dextran sulfate sodium (DSS) 3%. The colon was collected on the 6th day of colitis for histological analysis (a) in (b) immunophenotyping of CD11b^+^ cells, (c) dendritic proinflammatory cells (CD11b^+^CD11c^+^CD103^−^), and (d) tolerogenic dendritic cells (CD11b^+^CD11c^+^CD103^+^), evaluated by flow cytometry. (e) Myeloperoxidase (MPO) and (f) eosinophil peroxidase (EPO) assays. DSS: C57BL/6 mice exposed to DSS; ADX + DSS: C57BL/6 adrenalectomized mice exposed to DSS. The dotted lines correspond to the control group of C57BL/6 mice without colitis. Results are representative of two independent experiments, with 5 mice/group. ^*∗*^ *p* < 0.05.

Since the lack of adrenals clearly affected the pool of regulatory cells in the MLN but especially in the central compartment, the spleen, we aimed to investigate if the adrenal-derived mediators could differentially modulate the systemic and the local responses in the intestine during colitis induction. Histological analysis demonstrated that, in spite of the presence of adrenals, colitis mice had erosion areas of varying sizes in the colon, with injured surface epithelium and crypts, along with a loss of mucin and the presence of cuboid epithelial cells in the lesion borders. A predominance of mononuclear cells was found in the lamina propria of both colitis and colitis adrenalectomized groups. In the presence of adrenal glands mice showed mixed infiltrates of moderate-severe intensity in the submucosa and, surprisingly, the cellular infiltrate was of mild to moderate intensity in the absence of adrenals (Figure 3(a)), despite the systemic inflammation.

Therefore, we characterized some innate immune cells that could be associated with the constrained immune response in the gut after adrenalectomy (Supplementary Figure  1S in Supplementary Material available online at http://dx.doi.org/10.1155/2016/4936370). The diminished infiltrate in the absence of adrenals was related to a reduction in the frequency of CD11b^+^ leukocytes (Figure 3(b)) and CD11b^+^CD11c^+^CD103^−^ proinflammatory population of dendritic cells (Figure 3(c)) [22, 23]. Most interestingly, there was augmented frequency of the putative tolerogenic CD11b^+^CD11c^+^CD103^+^ dendritic cells in the gut of adrenalectomized mice exposed to DSS (Figure 3(d)), indicating a potential role for these antigen presenting cells in the constrained local response. On the other hand, there was no difference in the detection of myeloperoxidase and eosinophil peroxidase enzymes, which account for the activity of neutrophils and eosinophils, respectively, in these tissues (Figures 3(e) and 3(f)).

**Figure 4:**
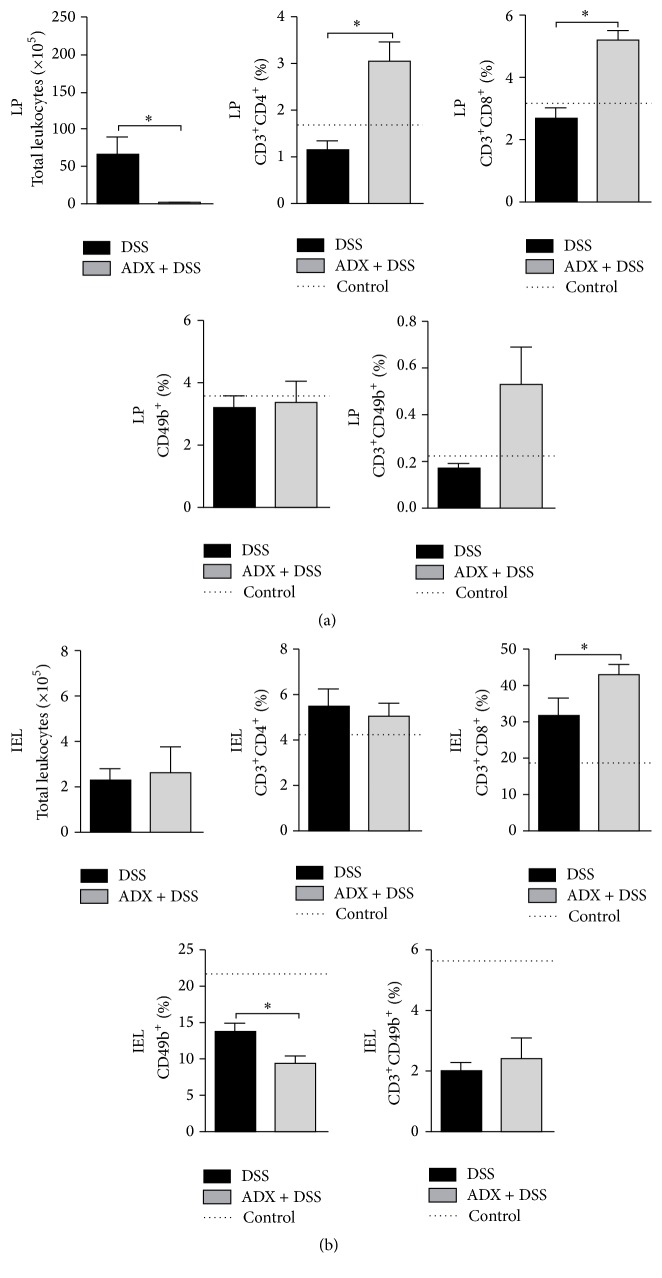
Adrenal-derived mediators altered the accumulation of lymphocytes in the intestine.C57BL/6 mice were adrenalectomized and exposed to dextran sulfate sodium (DSS) 3%. The colon was collected on the 6th day of colitis for phenotypic characterization of leukocytes from LP (lamina propria) (a) and IEL (intraepithelial cells) (b). After acquisition, samples were analyzed by FlowJo software. DSS: C57BL/6 mice exposed to DSS; ADX + DSS: C57BL/6 adrenalectomized mice exposed to DSS. The dotted lines correspond to the control group of C57BL/6 mice without colitis. Results are representative of two independent experiments, with 5 mice/group. ^*∗*^ *p* < 0.05.

Next, to further investigate if the adrenal-derived mediators also affected the accumulation of lymphocytes in the intestine, we quantified these populations in both lamina propria (LP) and intraepithelial lymphocytes (IEL) compartments of the colon (Supplementary Figure  2S). In fact, in accordance with histopathological data, there was a remarkable decrease in total leukocyte counts in the colon LP in the absence of adrenal glands (Figure 4(a)). Nevertheless, this reduced tissue infiltrate was composed by augmented frequency of T helper (CD3^+^CD4^+^), T cytolytic (CD3^+^CD8^+^), and NKT cells (CD3^+^CD49b^+^) after exposure to the colitis trigger, DSS (Figure 4(a)). In addition, the immunophenotypic analysis of IEL showed that absence of adrenals could be associated with a reduction in NK cells when compared to DSS nonadrenalectomized group, in contrast to augmented frequency of CD8 T cells in this gut compartment (Figure 4(b)). Altogether, these results suggested a role for adrenal glands and adrenal-derived hormones in the modulation of different cell populations that could influence inflammation control in the gut mucosa.

### 3.4. Adrenal-Derived Glucocorticoid Is One of the Key Players in the Control of Intestinal Inflammation

**Figure 5:**
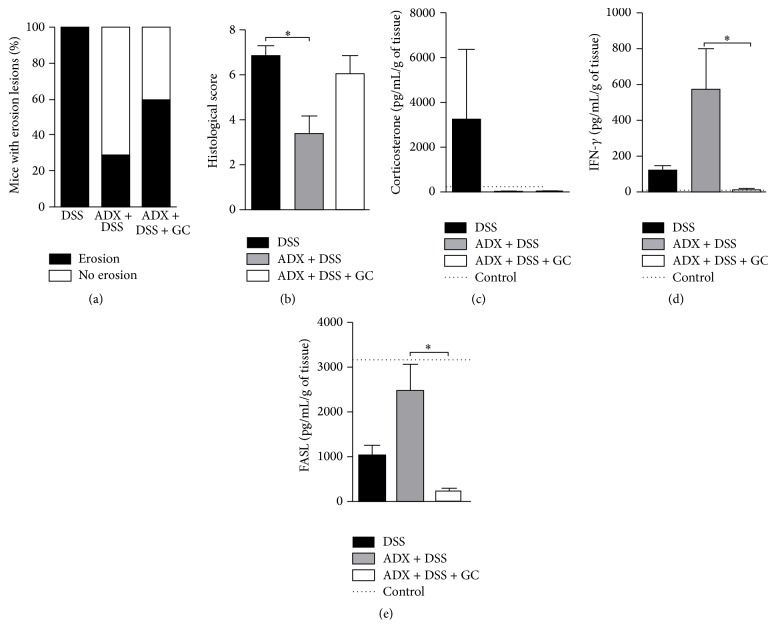
Adrenal-derived glucocorticoid is one of the key players in the control of intestinal inflammation. C57BL/6 mice were adrenalectomized and exposed to dextran sulfate sodium (DSS) 3%. After surgery, a group of mice was treated i.p. with 1 mg/Kg/day of dexamethasone from the 3rd to the 5th day of colitis, for replacement of GC, as described in Material and Methods. The colon was collected on the 6th day of colitis for histological analysis (a and b): (a) erosion lesions; (b) histological score; (c) intestinal corticosterone levels; (d) IFN-*γ*; and (e) FasL levels. DSS: C57BL/6 mice exposed to DSS; ADX + DSS: C57BL/6 adrenalectomized mice exposed to DSS. ADX + DSS + GC: C57BL/6 adrenalectomized mice exposed to DSS and treated with dexamethasone. The dotted lines correspond to the control group of C57BL/6 mice without colitis. Results are representative of two independent experiments, with 5 mice/group. ^*∗*^ *p* < 0.05.

Adrenals are responsible for the production of a series of hormones and neuroendocrine mediators, in special glucocorticoids (GC), which can modify the ongoing immune responses. Then, since removal of adrenal glands differentially impacted the mucosal immunity in the gut, we investigated the role of GC in this scenario, by treating adrenalectomized mice with corticoids. The frequency of mice with erosion areas after DSS exposure in the absence of adrenals was initially reduced but augmented after treatment with GC (Figure 5(a)). Most interestingly, the overall histological score that was diminished in adrenalectomized mice was restored to the same degree of that observed in DSS group, after GC replacement (Figure 5(b)), indicating that the main gut tissue alterations after adrenalectomy were dependent on the adrenal-derived GC. In fact, there was no compensatory local production of GC after adrenal removal (Figure 5(c)). In addition, there was augmented local IFN-*γ* production (Figure 5(d)), along with elevated proapoptotic FasL molecule on tissue homogenates, which were also dependent on adrenal GC (Figure 5(e)). These data suggested that the constrained gut inflammatory infiltrate after adrenalectomy could be a consequence of cytokine (IFN-*γ*) induced cell death mechanisms, such as FasL, aimed at reestablishing the gut homeostasis after barrier disruption and colitis induction. Taking together, these results pointed to GC as one of the key adrenal-derived hormones able to modulate intestinal inflammation.

## 4. Discussion

The results presented here showed that adrenal glands play divergent roles in the modulation of immune responses after triggering of intestinal inflammation. The main influence of these glands in experimental colitis seemed to be related to the activity of GC. This hypothesis was reinforced by the fact that mice treated with GC, in the absence of adrenal glands, had the local hallmarks triggered by colitis restored to the levels observed in adrenal-intact group.

In our study, gut inflammation led to the activation of adrenal glands, with a consequent increase in plasma levels of corticosterone, possibly in an attempt to constrain the exacerbated systemic inflammatory response. The adrenalectomized mice with colitis had increased IL-6 levels in contrast to lower LPS in serum. IL-6 is a proinflammatory cytokine produced by macrophages, T lymphocytes, and fibroblasts in response to bacterial infection. This molecule is also one of the main inflammatory mediators able to stimulate adrenal glands for GC production and subsequent inhibition of an ongoing immune response [24]. Most importantly, IL-6 may induce GC production by the hypothalamic-pituitary-adrenal axis or directly stimulate the adrenocortical cells with a later dampening of the inflammation [25]. So we suggested that disease worsening observed in adrenalectomized mice exposed to DSS could be related to higher levels of circulating IL-6 together with the absence of a counter regulation mediated by endogenous GC. In fact, it is of note that the clinical score depicted in our study comprised the sum of local and systemic signs that together contributed to the overall evaluation of mice. Therefore, while some parameters are specific to gut inflammation, such as wet anus, diarrhea, and bleeding stools, others may point to additional systemic dysfunctions, indicating further disease complication in mice without the adrenal glands.

The resulting immune reaction in pathophysiological conditions usually depends on the balance between effector cells producing inflammation and its modulation by regulatory mechanisms. Therefore, the failure observed in the control of systemic inflammation and disease worsening could be attributed to the reduced frequency of Tregs during exposure of adrenalectomized mice to DSS or to deficiencies in other regulatory mechanisms dependent on the adrenal glands. In this context, there was an overall decrease in the expression of several markers related to Tregs, which are known to have a significant ability to modulate the pathogenesis of many immune-mediated diseases [26]. The suppressive mechanisms of these cells may involve surface molecules such as CTLA-4 and PD-1, which are receptors that act synergistically and negatively regulate T cell activation [27]. CD73 is expressed on the surface of T lymphocytes and converts extracellular 5-AMP to adenosine. Thus, the adenosine inhibits the proliferation of effector cells and secretion of Th1 and Th2 cytokines [28]. Natural regulatory T cells or those induced by TGF-*β* express high levels of FR4 and the blockade of this receptor promotes the depletion of the regulatory cells [29, 30]. On the other hand, PD-1 is a cell surface receptor that triggers CD28 superfamily inhibitory pathways in order to attenuate the responses and promote T cell tolerance [31]. Dexamethasone increases PD1 expression in a dose-dependent manner and this effect may be completely inhibited by glucocorticoid receptor antagonist, indicating that the effect of GC on PD1 expression is mediated by the glucocorticoid receptor [32]. Likewise, FoxP3 is the master transcription factor of Tregs and its low expression or the absence of a functional molecule may lead to reduced suppression of exacerbated inflammatory responses, such as in IBD [33, 34]. Hence, the reduced frequency of regulatory cells in our study may be associated with the disease worsening in the absence of endogenous GC, since this hormone drives Tregs differentiation* in vivo* and* in vitro* [35, 36].

Interestingly, despite the overall anti-inflammatory potential of GC, adrenalectomized mice had reduced intestinal lesions, which could be due to the development of local regulatory mechanisms in an attempt to counteract the inflammation induced by DSS. This was evidenced by the increase of CD11c^+^CD11b^+^CD103^+^ dendritic cells (DC), which have a potentially tolerogenic profile [22], in contrast to a significant decrease of CD11c^+^CD11b^+^CD103^−^ proinflammatory DC [23]. Similarly, C57BL/6 mice deficient for CRH (CRH KO-*knockout*) exposed to TNBS had significant reduction of the intestinal inflammatory responses, lower levels of glucocorticoids, and increased IL-6 in the blood [37]. We also hypothesized that the local production of GC could be involved in the regulation of gut inflammation in mice with disrupted adrenal function. However, the extra adrenal production of this hormone was only detected in cultures of gut fragments obtained from mice with adrenal glands. Therefore, although the intestinal steroid genesis and GC synthesis may be induced by LPS in a TNF-*α*-dependent manner [18] and suppress intestinal inflammation [19], this phenomena did not seem to occur in our adrenalectomized mice, which presented low serum LPS on the time point evaluated. On the contrary, the local GC increase was dependent on adrenal-derived mediators, as observed in DSS group.

The severity of bowel inflammation is usually associated with elevated production of proinflammatory cytokines [38]. Here, the absence of adrenals led to levels of IFN-*γ* still higher than those observed in mice with colitis and intact adrenal glands, despite a diminished inflammatory infiltrate in the colon. In addition, the elevated levels of IFN-*γ* returned to normality after GC treatment, indicating that the regulation of the production of this inflammatory molecule in the gut is dependent on GC derived from adrenal glands. In accordance, mice that underwent TNBS-induced colitis followed by adrenalectomy had a marked increase in IFN-*γ* and IL-10 levels in serum [39]. Though similarities were observed between ours and Shibolet et al.'s study [39], this is the first time, to the best of our knowledge, that the impact of adrenal glands removal in colitis outcome was associated with the differential local modulation of the gut infiltrate, resulting in fewer intestinal lesions. Furthermore, our work also pointed to a relationship between adrenalectomy and the absence of systemic regulatory markers, which may elucidate the mechanisms that dictates the disease outcome. Accordingly, animals subjected to sepsis in the absence of the adrenals had much higher mortality and elevated concentrations of the other inflammatory cytokine IL-17 [40]. It is largely known that the excessive production of both cytokines, IL-17 and IFN-*γ* by Th1 and Th17 cells, respectively, is involved in the pathogenesis of IBD [41, 42]. Therefore, besides their inflammatory potential, we suggested that the Th1 or CD3^+^CD8^+^ cells in the inflamed mucosa of ADX + DSS mice may act as one of the main players protecting them against pathogens translocation to the mucosa after barrier disruption. IFN-*γ* is the major physiological activator of macrophages, which, when activated, produce cytokines such as IL-1, TNF, and IL-6 that contribute to inflammation [43]. IFN-*γ* is also capable of inducing the expression of MHC-II molecule in intestinal epithelial cells (IEC), allowing the presentation of microbial antigens to immune competent cells. This increase in MHC-II expression in response to IFN-*γ* may be markedly inhibited by dexamethasone [44]. In addition, in our study, the decreased cellularity in the intestinal mucosa of adrenalectomized mice exposed to DSS was associated with FasL-mediated apoptosis in parallel to the increase of IFN-*γ*, in a GC-dependent manner. The binding of FasL to its receptor (FasR or CD95) induces apoptosis in sensitive cells [45]. Apoptosis induced by Fas plays a fundamental role in the maintenance of immunological tolerance and is involved in cytotoxic activity of T cells [46] and NK cells [47]. Likewise, after the elimination of invading pathogens, apoptosis controls innate and adaptive immune responses in order to reestablish immune homeostasis, thus preventing host tissue damage caused by excessive inflammation [48, 49]. Then, we suggested that IFN-*γ* may have been produced to control local microbiota translocation and, together with infection control, may have induced FasL with a consequent reduction of gut cellularity by apoptosis mechanisms. Indeed, IFN-*γ* plays an important role in apoptosis induction or reduction of CD4^+^ T cells during infectious diseases [50, 51], in response to tumors [52] and in the experimental autoimmune encephalomyelitis [53]. On the other hand, GCs present an outstanding anti-inflammatory potential and the control of immune responses in mice submitted to adrenalectomy and treated with exogenous GC may have facilitated bacteria translocation from the gut, once the inflammation is essential to control the local microbiota. It is possible that, in adrenalectomized mice (ADX + DSS group), which are devoid of systemic regulation by GC, the immune response in the gut was strong and fast enough to control bacteria translocation from the lumen, thus avoiding a continuing or chronic inflammation. Although future studies are still necessary to prove this hypothesis, we believe that these mice might have developed local mechanisms (such as FasL production), raised in an attempt to constrain the triggered immune response and finalize the local reaction, which probably peaked previously in the absence of adrenals (data not investigated in this paper). Furthermore, it is essential to emphasize that the responses in the gut mucosa must be always fine-tuned to avoid excessive inflammation while still being able to control invading pathogens. Then, any disturbance in this homeostasis may facilitate the development of inflammatory bowel diseases.

Historically, it is known that the mortality rate of patients with adrenal insufficiency was considerably high before the use of GC [54]. The deficiency in GC production is clinically associated with impaired resistance to stress, lymphoid tissue hypertrophy, weight loss, and hypoglycemia [55–57]. Then, in addition to suppressing the inflammatory response, the GC is also responsible for increased blood glucose concentration and various effects on the metabolism of carbohydrates, proteins, and lipids [58–60]. Based on this, we cannot exclude the involvement of metabolic alterations in disease worsening in the absence of adrenals nor the role of other mediators than GC commonly produced by these glands.

In summary, our results showed that the adrenal glands play divergent roles in the control of local and systemic inflammation during breakdown of mucosal tolerance. The lack of a counter regulatory hormone together with reduced frequency of Tregs probably led to impaired control of systemic inflammatory reaction. On the other hand, in the absence of endogenous GC, the intestine developed specific modulatory mechanisms able to control the local cytokine production and leukocyte accumulation, which culminated in more constrained tissue damage. Nevertheless, although this local response is enough to control gut commitment in colitis, it is not sufficient to prevent host susceptibility to systemic inflammation raised in the absence of adrenal glands. Finally, this study brings new features for a better understanding of the relationship between the immune and endocrine systems, providing basis for development of future therapies for patients with Crohn's disease or ulcerative colitis.

## Supplementary Material

In order to identify the profile of innate immune cells in gut, the populations of CD11b+ cells (Fig. 1S B), inflammatory and tolerogenic dendritic cells (CD11b+CD11c+CD103- and CD11b+CD11c+CD03+, respectively – Fig. 1S C) were characterized in lamina propria (LP) compartment.
