# Supplementary material for: Adrenal-Derived Hormones Differentially Modulate Intestinal Immunity in Experimental Colitis
Source: Mediators Inflamm. 2016 Jun 14;2016:4936370. doi: 10.1155/2016/4936370 (PMC4923585; doi:10.1155/2016/4936370)
Supplement: Supplementary file 1 — In order to identify the profile of innate immune cells in gut, the populations of CD11b+ cells (Fig. 1S B), inflammatory and tolerogenic dendritic cells (CD11b+CD11c+CD103- and CD11b+CD11c+CD03+, respectively – Fig. 1S C) were characterized in lamina propria (LP) compartment. [file 4936370.f1.docx]

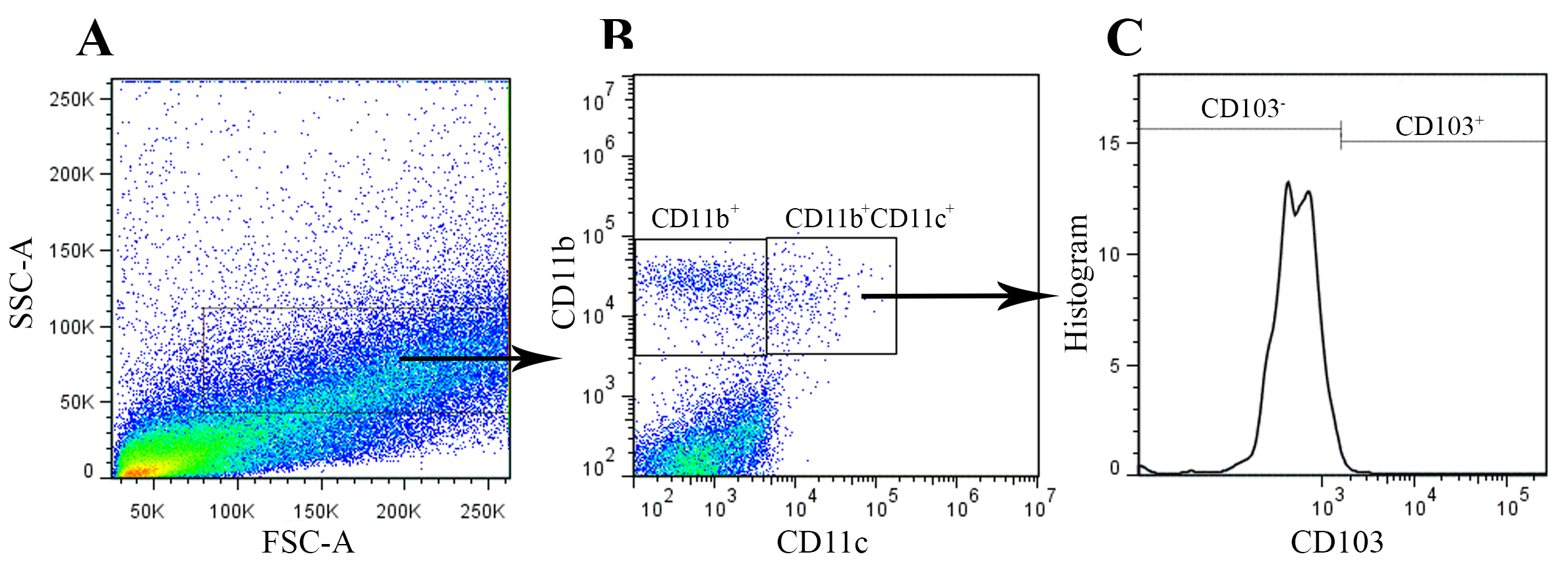


**Figure 1S.** Gating strategy for evaluation of CD11b^+^ cells, inflammatory and tolerogenic dendritic cells (CD11b^+^CD11c^+^CD103^-^ and CD11b^+^CD11c^+^CD03^+^, respectively) in lamina propria (LP) compartment. Leukocytes were obtained as described in *Material and Methods* and processed for flow cytometry. After acquisition, data were analyzed using Flow Jo software. In (A), monocytes gating according to cell size (FSC) and granulosity (SSC), followed by dot plots and gating on CD11b^+^ or CD11b^+^CD11c^+^ stained cells (B). The frequency of CD103^+^ or CD103^-^ dendritic cells was verified by histogram in the double positive CD11bCD11c population, as in (C).


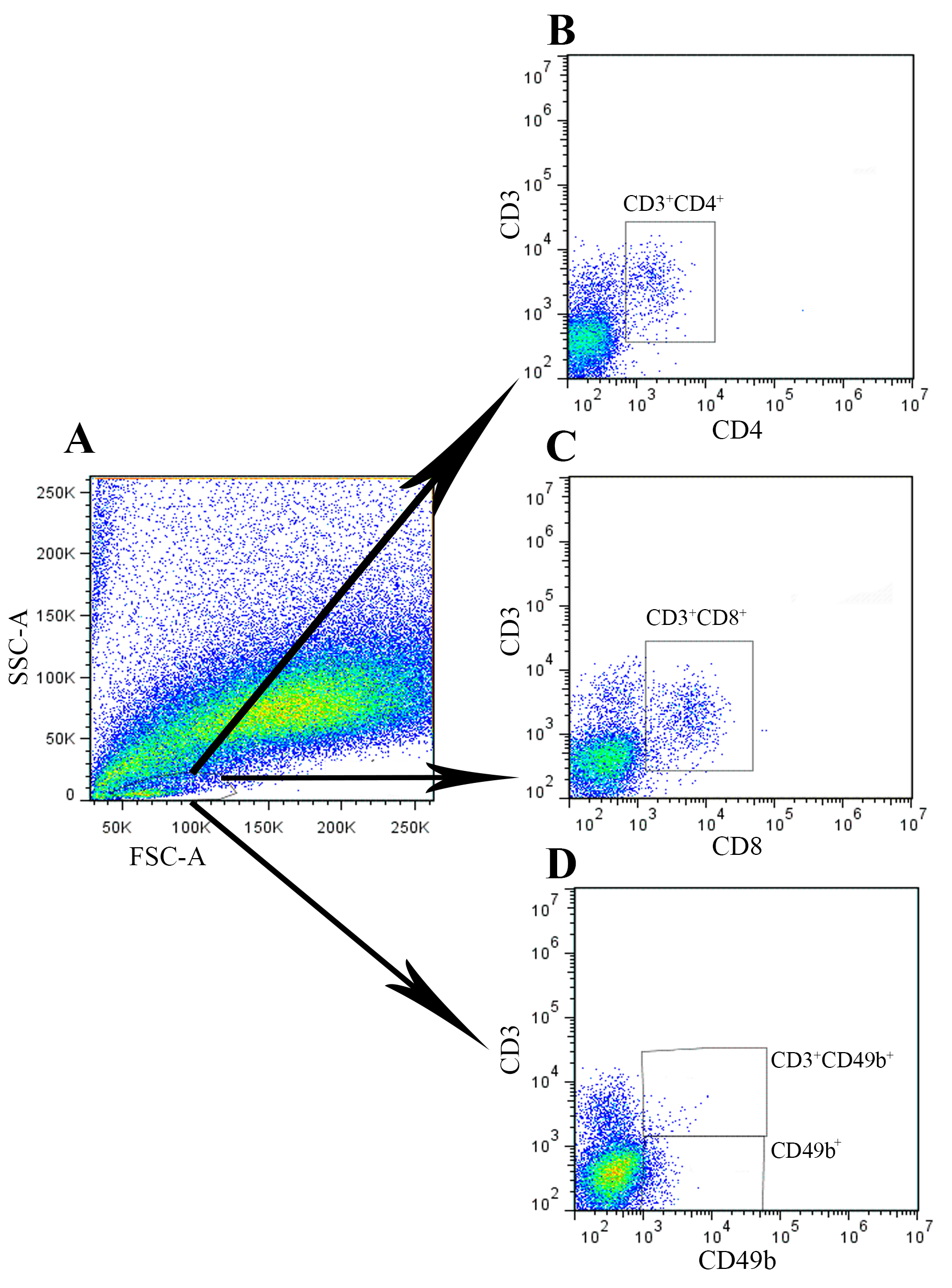


**Figure 2S.** Gating strategy for evaluation of TCD4 (CD3^+^CD4^+^), TCD8 (CD3^+^CD8^+^), NK (CD49b^+^) and CD3^+^CD49b^+^ (supposedly NKT cells), in lamina propria (LP) compartment and in intraepithelial lymphocytes (IEL) region of the colon. Leukocytes were obtained as described in *Material and Methods* and processed for flow cytometry. After acquisition, data were analyzed using Flow Jo software. In (A), lymphocytes gating according to cell size (FSC) and granulosity (SSC), followed by dot plots and gating on CD3^+^CD4^+^ (B), CD3^+^CD8^+^ (C), CD49b^+^ and CD3^+^CD49b^+^ population (D), for determination of lymphocytes frequency.
